# Biologic markers of risk in nipple aspirate fluid are associated with residual cancer and tumour size

**DOI:** 10.1038/sj.bjc.6690832

**Published:** 1999-12

**Authors:** E R Sauter, H Ehya, J Babb, E Diamandis, M Daly, A Klein-Szanto, E Sigurdson, J Hoffman, J Malick, P F Engstrom

**Affiliations:** Division of Population Science Departments of Pathology andSurgery, Fox Chase Cancer Center, Philadelphia, PA 19111, USA; Department of Pathology and Laboratory Medicine, Mount Sinai Hospital, Toronto, Ontario, Canada M5G 1X5

**Keywords:** nipple aspirate fluid, mastectomy, biomarkers

## Abstract

We previously demonstrated that nipple aspirate fluid (NAF) can be obtained from virtually all non-Asian women between the ages of 30 and 72. The focus of this report is to (1) determine the association of candidate markers of breast cancer risk in NAF obtained from fresh mastectomy specimens with residual breast carcinoma, and (2) evaluate the association of the markers with breast tumour progression. Nipple aspiration was performed on 97 specimens. Cytology, DNA index (including % hypertetraploid cells), cell cycle parameters (S phase fraction, % cells in G2/M), prostate-specific antigen (PSA), epidermal growth factor (EGF), testosterone, carcinoembryonic antigen (CEA) and prostaglandin D synthase (PGDS) were evaluated in NAF for their association with (1) residual ductal carcinoma in situ (DCIS) or invasive cancer, and (2) pathologic tumour size. NAF was obtained from 99% (96/97) of specimens. Atypical and malignant NAF cytology were significantly associated with residual DCIS or invasive cancer (*P* = 0.001) and with larger tumours (*P* = 0.004). One hundred per cent and 88% of subjects with malignant and atypical NAF cytology, respectively, had residual carcinoma. The percentage of cells in G2/M and DNA index were associated both with risk of residual carcinoma (*P* = 0.01 for each) and larger tumour size (DNA index, *P* = 0.03; G2/M, *P* = 0.05), although neither biomarker improved the ability of NAF cytology, to predict residual breast cancer. Higher DNA index was associated with atypical cytology (*P* = 0.0001). In summary, atypical and malignant NAF cytology are associated with larger tumour size, and are highly predictive of residual carcinoma after needle or excisional biopsy of the breast. © 1999 Cancer Research Campaign


*British Journal of Cancer* (1999) **81**(7), 1222–1227
© 1999 Cancer Research Campaign
Article no. bjoc.1999.0832

### Biologic markers of risk in nipple aspirate fluid are associated with residual cancer and tumour size

**ER Sauter[1], H Ehya[2], J Babb[1], E Diamandis[4], M Daly[1], A Klein-Szanto[2], E Sigurdson[3], J Hoffman[3], J Malick[1] and PF Engstrom[1]**

[1]Division of Population Science and the Departments of [2]Pathology and [3]Surgery, Fox Chase Cancer Center, Philadelphia, PA 19111, USA; [4]Department of Pathology and Laboratory Medicine, Mount Sinai Hospital, Toronto, Ontario, Canada M5G 1X5

**Summary** We previously demonstrated that nipple aspirate fluid (NAF) can be obtained from virtually all non-Asian women between the ages of 30 and 72. The focus of this report is to (1) determine the association of candidate markers of breast cancer risk in NAF obtained from fresh mastectomy specimens with residual breast carcinoma, and (2) evaluate the association of the markers with breast tumour progression. Nipple aspiration was performed on 97 specimens. Cytology, DNA index (including % hypertetraploid cells), cell cycle parameters (S phase fraction, % cells in G2/M), prostate-specific antigen (PSA), epidermal growth factor (EGF), testosterone, carcinoembryonic antigen (CEA) and prostaglandin D synthase (PGDS) were evaluated in NAF for their association with (1) residual ductal carcinoma in situ (DCIS) or invasive cancer, and (2) pathologic tumour size. NAF was obtained from 99% (96/97) of specimens. Atypical and malignant NAF cytology were significantly associated with residual DCIS or invasive cancer ($P = 0.001$) and with larger tumours ($P = 0.004$). One hundred per cent and 88% of subjects with malignant and atypical NAF cytology, respectively, had residual carcinoma. The percentage of cells in G2/M and DNA index were associated both with risk of residual carcinoma ($P = 0.01$ for each) and larger tumour size (DNA index, $P = 0.03$; G2/M, $P = 0.05$), although neither biomarker improved the ability of NAF cytology, to predict residual breast cancer. Higher DNA index was associated with atypical cytology ($P = 0.0001$). In summary, atypical and malignant NAF cytology are associated with larger tumour size, and are highly predictive of residual carcinoma after needle or excisional biopsy of the breast. © 1999 Cancer Research Campaign

**Keywords:** nipple aspirate fluid; mastectomy; biomarkers

After a diagnosis (by fine needle, core needle, incisional or excisional biopsy) of in situ or invasive breast cancer, thousands of women undergo mastectomies each year in order to obtain optimal local control of their disease. Two of the primary reasons for performing mastectomy are large tumour size and inability to obtain negative tumour margins after one or more excisional biopsies. Many women presumed to have large tumours by clinical assessment undergo mastectomy after diagnosis by fine or core needle aspiration. Unfortunately, clinical assessment may under- or overestimate actual tumour size (Harris et al, 1993).

In the recently operated breast, mammography and breast examination are generally of little help in predicting residual disease. A number of pathologic factors in the diagnostic biopsy have been associated with residual disease/local failure in the breast, including positive margins, gross multicentricity, extensive intraductal carcinoma, age under 35–40, and invasive lobular carcinoma (Lagios, 1992; Harris et al, 1993). Despite the currently available markers, approximately half of the women who undergo mastectomy for presumed residual disease will not have disease when the breast is investigated microscopically. Additional markers are needed to better tailor therapy for these women.

Present efforts to evaluate the breast directly either through evaluation of tissue or individual cells have been hindered because the analysis of these specimens generally required an invasive procedure. The adult, non-pregnant, non-lactating breast secretes fluid into the breast ductal system. This fluid can be obtained through aspiration of the nipple with a modified breast pump. Refinements in the ability to obtain this fluid, as well as epidemiologic studies to identify subjects most likely to yield NAF, have been ongoing for over 20 years. Nipple aspiration has the attractiveness of quickly, painlessly, and non-invasively obtaining both breast epithelial cells (the cells at risk for transformation to breast cancer), as well as secreted proteins, which are concentrated in the fluid. We are now able to obtain cellular NAF samples (≥ ten breast epithelial cells on a slide) in the majority of subjects (Sauter et al, 1997). Those samples containing few or no breast epithelial cells are also informative, for we have shown that NAF cytology of low cellularity is associated ($P = 0.001$) with low breast cancer risk (Sauter et al, 1997). We have also demonstrated that secreted proteins in NAF, such as prostate-specific antigen (PSA) (Sauter et al, 1996), can be analysed and are associated ($P = 0.002$) with risk.

The goal of this study was to determine if biological markers of breast cancer risk in NAF from mastectomy specimens were associated with residual disease in the breast and/or primary tumour size. We performed nipple aspiration on fresh mastectomy specimens rather than on subjects prior to mastectomy for practical reasons. There is generally only a short interval of a few days to a few weeks between the time of breast cancer diagnosis and

*Received 3 September 1998*
*Revised 31 March 1999*
*Accepted 27 April 1999*

*Correspondence to:* ER Sauter

Presented in part at the American Association for Cancer Research, New Orleans, LA in March 1998.

mastectomy, so an additional visit to the clinic for breast aspiration prior to mastectomy may be difficult. The subjects to be aspirated are very anxious due to their recent diagnosis of breast cancer, are already required to undergo a number of clinical tests (blood tests, X-rays, etc.) and may be reluctant to undergo an additional test with unproven benefit. By aspirating the breast immediately after removal we avoided these potential problems.

Each of the markers chosen for analysis has proven or suspected importance in breast cancer. We have previously demonstrated that NAF cytology and PSA levels in NAF are associated with breast cancer risk (Sauter et al, 1996, 1997). EGF stimulates breast cancer cell growth (Earp et al, 1995). CEA is frequently found in subjects with metastatic breast cancer and, in combination with other markers, is useful in predicting breast cancer recurrence (Leis, 1991). Changes in DNA content and cell cycle parameters, including DNA index, S phase fraction, and G2/M, are breast cancer prognostic markers frequently used in clinical practice (Yeatman and Bland, 1991). PGDS is an enzyme recently found in breast cyst fluid and in breast tumours, with potential as a marker of breast cancer risk (Melegos et al, 1991). If markers in NAF predictive of residual cancer and/or of tumour size are identified, they may prove useful to help guide therapy regarding further surgery and/or radiation therapy.

### MATERIALS AND METHODS

### Subjects

Ninety-seven specimens from 95 subjects aged 30–79 years (median 52 years) were collected for the study between January 1995 and July 1997 after approval of the Fox Chase Institutional Review Board. Eighty-one per cent of the subjects were white, 15% African-American and 4% Hispanic. Each breast had undergone needle or excisional biopsy demonstrating ductal carcinoma in situ (DCIS) or invasive cancer. A mastectomy was performed a median of 22 days (range 2–84 days) after excisional biopsy, with the exception of three subjects who received neoadjuvant chemotherapy prior to mastectomy. NAF was obtained from 96/97 specimens. Mastectomy specimens were categorized as having residual cancer if either invasive carcinoma (IC) or DCIS was present in the breast, or no residual cancer if neither IC or DCIS was present. (This category included subjects whose specimens contained lobular carcinoma in situ (LCIS), atypical hyperplasia (AH), hyperplasia without atypia, or normal breast tissue.) Subjects were also grouped by the method of disease diagnosis: needle biopsy (either fine or core) or excisional biopsy. No subject underwent incisional biopsy. If an individual underwent both needle and excisional biopsy prior to mastectomy, they were placed in the excisional biopsy group.

The primary indication for mastectomy for each of the subject specimens is outlined in Table 1. For subjects diagnosed by needle biopsy (NB), all were presumed to have residual disease. Sixteen of 24 (67%) subjects diagnosed by NB underwent mastectomy rather than excisional biopsy due to large tumour size, while subject preference was the primary reason for mastectomy rather than excisional biopsy in the other eight subjects. The presence of breast cancer at or near the surgical margin, or an unknown margin status, was the deciding factor in each of the subjects who underwent mastectomy after excisional biopsy. After excisional biopsy, margins were involved in 52/72 (72%), close in 18/72 (25%), or unknown in 2/72 (3%). Pathology records were also reviewed for the presence in each breast cancer of an extensive intraductal component, for multifocality and for multicentricity.

**Table 1** Primary indication for mastectomy provided by the clinical record

| Indication | Needle biopsy | Excisional biopsy |
|---|---|---|
| Carcinoma size | 18 | 0 |
| Patient preference | 6 | 0 |
| Involved margin | NA | 52 |
| Close (< 2 mm) margin | NA | 18 |
| Unknown margin | NA | 2 |

NA: not applicable.

### Aspiration technique

Nipple fluid was aspirated by a trained physician or nurse clinician using a modified breast pump (Sauter et al, 1996). The mastectomy specimen was transported from the operating room to the histology facility immediately after resection. The breast nipple was cleansed with alcohol, the plunger of the aspiration device was withdrawn to the 7 ml level and held for 15 s. Fluid in the form of droplets was collected in capillary tubes. The quantity of fluid varied from 1 µl to 200 µl.

If keratin plugs rather than NAF were obtained after suction was completed, the plugs were removed with an alcohol swab and suctioning repeated. On some occasions, this procedure was repeated two or three times to remove the plugs before fluid was obtained. In order to obtain additional fluid, the nipple was gently compressed. One or two additional droplets of fluid often appeared.

### Cytology

#### *Specimen preparation*

The NAF was collected in 50-µl capillary tubes and rinsed into a container with 1 ml of 3% polyethylene glycol in ethanol–isopropanol. The specimen was then cytocentrifuged onto ten glass slides. Three of the slides were used for cytological examination. If the slides contained < 10 epithelial cells, two additional slides were examined. The remaining slides (five or seven) were stored for biomarker studies. The slides selected for cytological examination were washed twice in 95% ethanol for 10 min each, rehydrated in tap water and stained by the Papanicolaou method.

#### *Specimen interpretation*

The Papanicolaou-stained smears were examined by a cytopathologist (HE) experienced with breast cytology. The examiner was not aware of the histology of each specimen. Moreover, since the NAF specimens analysed in this study were mixed with specimens from subjects with an intact breast, the examiner was not aware of which NAF specimens were from mastectomies. Each specimen was designated as containing scant epithelial cells (class I), normal epithelial cells (class IIA), hyperplasia without atypia (class IIB), atypia (class III), or malignant cells (class IV), using criteria previously described (Sauter et al, 1997). This is a modification of terminology used by King et al (1983), and is to be differentiated from the Papanicolaou classification.

© 1999 Cancer Research Campaign

### Histology

### *Specimen preparation*

After breast aspiration was completed, the mastectomy specimen was fixed in 10% neutral buffered formalin for 16–24 h and embedded in paraffin wax. Eighteen to 20 representative blocks were prepared, and one 5-μm slide per block was cut and stained with haematoxylin and eosin (H&E).

### *Specimen interpretation*

Histological review of each H&E slide from the needle aspiration, excisional biopsy and mastectomy was performed. Margin status was assessed in the excisional biopsies. A margin was defined as positive if tumour was present at the margin, close if tumour was present ≤ 2 mm from the margin, and negative if present > 2 mm from the margin.

### Image analysis

Only specimens with adequate cellularity (≥ 10 breast epithelial cells on Papanicolaou-stained slides, cytology classes II–IV) were evaluated by image analysis. Sixty-one (64%) specimens were sufficiently cellular to perform image analysis.

### *Specimen preparation*

A standardized quantitative DNA staining kit (RIAS Feulgen Stain Kit, Roche Image Analysis Systems, Elon College, NC, USA) was used following the manufacturer's instructions. In brief, after rehydration the slides were processed for hydrolysis with 5 N hydrochloric acid for 60 min and then transferred to the staining solution (Schiff's reagent) for 1 h, rinsed, dehydrated and mounted with synthetic resin.

### *Specimen interpretation*

The Roche Pathology Workstation (Ellison et al, 1995) was used to evaluate nuclear ploidy, S phase fraction, % cells in G2/M and % hypertetraploid cells (cells with greater than twice their complement of DNA). Human lymphocytes were used as a control diploid cell population. All epithelial cells if under 100, or a minimum of 100 cells if more were present on a slide, were measured per case and type of stain. The average number of cells counted per specimen was 30.

### PSA, PGDS, EGF, CEA, testosterone

NAF was extracted from glass capillaries as previously described (Sauter et al, 1996). The sample was analysed for total protein with the bicinchoninic acid method (Pierce Chemical Co., Rockford, IL, USA).

### *PSA and PGDS*

Both candidate markers in NAF were analysed using a highly sensitive and specific immunofluorometric procedure (Melegos et al, 1991; Sauter et al, 1996). The assays use mouse monoclonal anti-PSA capture antibodies coated to polystyrene microtitre wells, a biotinylated monoclonal detection antibody, and alkaline phosphatase-labeled streptavidin (SA-ALP). For each assay, 100 μl of sample is incubated with the coating antibody in the presence of 50 μl of assay buffer containing the detection antibody. After incubation for 1 h and washing × 6, the SA-ALP conjugate is added for 15 min, followed by washing × 6. The activity of ALP is then measured by adding the substrate 5-fluorosalicylphosphate, incubating for 10 min, and by then adding an EDTA-$Tb^{3+}$ solution to form a ternary fluorescent complex between the released 5-fluorosalicylate, $Tb^{3+}$ and EDTA. The fluorescence is measured in the time-resolved fluorometric mode.

### *EGF, CEA, testosterone*

Epidermal growth factor concentration was determined by RIA as previously described (Lai et al, 1989). The sensitivity of the assay was 0.04 ng $ml^{-1}$. CEA was measured by RIA as previously described (Thompson et al, 1969). Testosterone was quantified first by extraction with hexane:ethylacetate and subjection to Celite column partition chromatography prior to RIA (Falk et al, 1996).

### Statistical analysis

Spearman's rank correlation coefficient and Kendall's tau were used to determine if there was an association between risk of residual cancer in the mastectomy specimen and a variety of candidate markers in NAF: cytology, DNA index (including % hyper-tetraploid cells), cell cycle parameters (S phase fraction, % cells in G2/M), PSA, EGF, testosterone and PGDS. Samples were considered together or by method of diagnosis, and risk of residual cancer was categorized as either no cancer or residual cancer (DCIS or invasive cancer). Step-wise logistic regression models (Agresti, 1990) were fit to the data to determine whether increasing breast cancer risk categories were associated with cytological grouping, DNA index, and/or % cells in G2/M. Multiple risk categorization models were evaluated.

The association between pathologic tumour size and (1) NAF cytology and (2) method of diagnosis were examined using the $\chi^2$ test for independence. Differences in quantitative biomarker expression (PSA, G2/m, EGF, DNA index, hypertetraploidy, CEA, PGDS, S phase fraction and testosterone) among the specimen groups defined by tumour size were examined using both parametric (*t*-test, analysis of variance) and non-parametric (Wilcoxon rank sum and Kruskal–Wallis) test procedures.

### RESULTS

The association of residual cancer with a number of candidate markers of breast cancer risk: S phase fraction, % cells in G2/M, % hypertetraploid cells, PSA, CEA, EGF, testosterone and PGDS was evaluated. The markers were initially evaluated separately based on the method of biopsy, for all 24 specimens in the needle biopsy group contained residual in situ or invasive cancer within the breast (lymph node status was not considered), as opposed to 54% (39/72) of mastectomy specimens after excisional biopsy. No significant differences were identified for any of the markers based on biopsy method with the exception of cytology (Table 2). The difference in NAF cytology based on diagnostic method was not detected when NAF cytology was compared only in mastectomy specimens which contained residual cancer (DCIS or invasive cancer). When samples from both diagnostic groups were combined and categorized as containing no cancer or residual cancer, the presence of residual cancer was significantly associated with abnormal cytology ($P$ = 0.001), % cells in G2/M and DNA index (both $P$ = 0.01). DNA index was also associated with NAF cytology ($P$ = 0.0001).

 *© 1999 Cancer Research Campaign*

**Table 2** Nipple aspirate fluid cytology obtained from mastectomy specimens

| Marker | NB | Ex biopsy[1] | *P*-value |
|---|---|---|---|
| Mastectomy diagnosis | | | |
| Normal, AH, LCIS[2] | 0, 0, 0 | 25, 4, 3 | |
| DCIS[2] | 1 | 16 | |
| Invasive cancer | 23 | 24 | 0.0001 |
| Cytology (all specimens) | | | |
| I–II | 13 (54%) | 57 (79%) | |
| III–IV | 11 (46%) | 15 (21%) | 0.01 |
| Cytology (subjects with residual DCIS or invasive cancer in the mastectomy specimen) | | | |
| I–II | 13 (54%) | 25 (64%) | |
| III–IV | 11 (46%) | 14 (36%) | 0.36 |

NB, needle biopsy; Ex biopsy, excisonal biopsy; AH, atypical hyperplasia; LCIS, lobular carcinoma in situ; DCIS, ductal carcinoma in situ.

**Table 3** Sensitivity and specificity of nipple aspirate cytology after excisional biopsy

| | Residual cancer | |
|---|---|---|
| Nipple aspirate cytology | Yes | No |
| III or IV | 14 | 1 |
| I or II | 25 | 32 |
| All | 39 | 33 |

A number of histological characteristics were evaluated for their association with abnormal (class III or IV) NAF cytology. Neither extensive intraductal component (EIC) nor multifocality and/or multicentricity (mf/mc) was significantly associated with abnormal NAF cytology after excisional biopsy. Abnormal NAF cytology was found in 24% of breast cancers with EIC and in 22% of cancers with mf/mc compared to 19% of cancers without EIC and 20% of cancers without mf/mc.

The ability of abnormal (class III–atypical, or class IV–malignant) NAF cytology to predict the presence of residual DCIS or invasive cancer (residual cancer) was next evaluated. Subjects were divided by method of diagnosis, for all mastectomy specimens after needle biopsy alone had residual cancer, as opposed to only 56% after excisional biopsy. Since all mastectomy specimens from subjects who had undergone needle biopsy alone had residual cancer, the sensitivity of NAF cytology could not be evaluated. Eleven of 24 (46%) mastectomies performed after needle biopsy alone contained class III or IV NAF cytology (Table 2). When mastectomies performed after excisional biopsy were evaluated, NAF cytology (class I, II vs class III, IV) was 97% specific (32/33 specimens) in ruling out residual cancer, with the only false positive coming from a class III cytology specimen (Table 3, Figure 1). On the other hand, NAF cytology was only 36% sensitive (14/39 specimens) in detecting residual cancer.

Candidate markers in NAF were also evaluated (Table 4) after specimens were categorized by pathological tumour size (DCIS + $T_1$ vs $T_{2–4}$). The T size reflects the best estimate of tumour diameter considering all diagnostic procedures and the mastectomy specimen. Larger tumours were significantly associated with (Table 4, Figure 2): atypical cytology ($P$ = 0.004), disease diagnosed by needle biopsy ($P$ = 0.002), higher DNA index ($P$ = 0.03) and % cells in G2/M ($P$ = 0.05).

When evaluated individually via logistic regression, NAF cytology, DNA index and % cells in G2/M were found to be significantly associated with the risk of residual cancer. Forward selection stepwise logistic regression was implemented to identify a model based on these three markers which best predicted the presence of residual cancer in the breast. This resulted in a model based only on NAF cytology. Thus, the addition of information on DNA index and/or % cells in G2/M did not significantly improve the ability to predict residual cancer once NAF cytology was included in the model.

### DISCUSSION

When we initiated this study, we wished to identify markers in NAF which were associated with residual cancer in the breast, as well as with tumour size. Because of the difference in the presence of as well as the quantity of residual tumour in the needle biopsy and excisional biopsy mastectomy specimens, we felt it important to analyse the groups separately. As expected, 100% of the mastec-

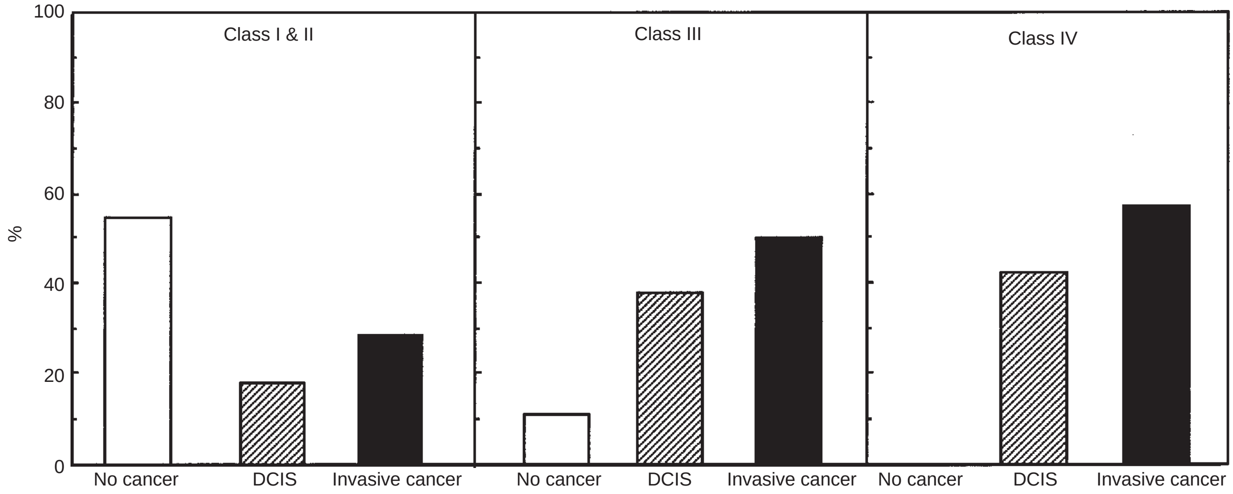

**Figure 1** Association of nipple aspirate cytology (class I–IV) with residual DCIS or invasive cancer after excisional biopsy

**Table 4** Association of biomarkers with primary tumour size[a]

| | Specimens | (Tis, $T_1$)$^2$ | ($T_{2–4}$)$^2$ | *P*-value |
|---|---|---|---|---|
| Cytology | | | | |
| I, IIA, IIB | 69 | 39 | 30 | |
| III, IV | 26 | 6 | 20 | 0.004 |
| Median % Cells in G2/M | 63 | 1.79 | 5.07 | 0.05 |
| Median DNA index | 63 | 1.11 | 1.31 | 0.03 |

[a]Tumour size: includes subjects diagnosed by either needle or excisional biopsy. (Tis, $T_1$, $T_{2–4}$)$^2$: Tis-carcinoma in situ; $T_1$: tumour ≤ 2 cm; $T_{2–4}$: tumour > 2 cm and/or with direct extension to the chest wall or skin. Complete TNM information was not available for one specimen.

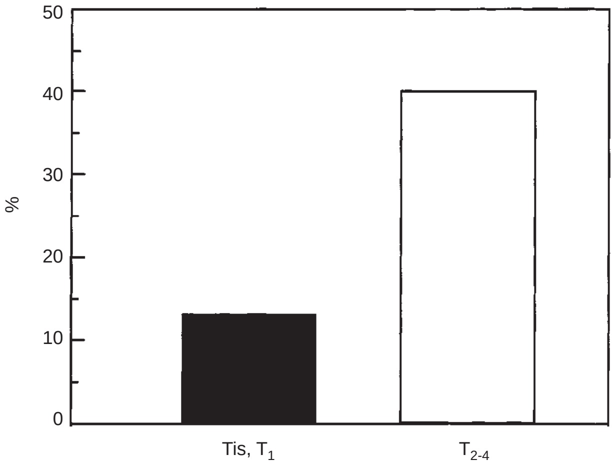

**Figure 2** Percentage of specimens with abnormal (class III–IV) nipple aspirate cytology based upon primary tumour size tomies performed after needle biopsy alone had residual DCIS or invasive cancer, whereas only 39/72 (54%) mastectomies performed after excisional biopsy contained residual DCIS or invasive cancer. When separated by method of diagnosis, none of the biomarkers were significantly different. For this reason, we did not separate the groups when evaluating the association of biomarkers with primary tumour size.

When an individual is diagnosed with breast cancer after needle biopsy, the decision to proceed to mastectomy rather than excisional biopsy is often based on clinical tumour size, although the estimation may be different from the actual (pathologic) size (Harris et al, 1993). After excisional biopsy, many women undergo mastectomy for presumed residual cancer, although controversy remains regarding the necessity to obtain microscopically clear margins (Hallahan et al, 1989; Veronesi et al, 1990; Sauer et al, 1992, Anscher et al, 1993). Thousands of women who undergo mastectomy after excisional biopsy will not have in situ or invasive cancer found in the mastectomy specimen. The chance of finding residual cancer is related to tumour margin status in the diagnostic specimen. If the margin is close, the likelihood is 23–32%; if positive, 53–65%; if unknown, approximately 45%, and if negative, approximately 26% (Frazier et al, 1989; Gwin et al, 1993). It is not unexpected that some subjects with negative margins will have residual cancer, given the possibility of multifocal and/or multicentric disease. Others have found residual tumour in 32–63% of reexcisional biopsies and mastectomies (Schwartz et al, 1984; McCormick et al, 1987; Schnitt et al, 1987; Olivotto et al, 1989; Stotter et al, 1989).

Invasive breast cancer develops at or near the original biopsy site in over 25% of patients with DCIS, whereas DCIS is uncommon (0.2–6%) in randomly selected breasts from autopsy series (Harris et al, 1993). On the other hand, LCIS left untreated leads to an increased risk of invasive carcinoma equally distributed in either breast (Haagensen et al, 1978). These data suggest that DCIS is a precursor to invasive breast cancer in the area of the in situ disease, whereas LCIS is a marker of increased risk in either breast. For the purposes of our analysis, LCIS was not considered residual cancer if found in the mastectomy specimen.

The breast ducts of adult nonpregnant women secrete small amounts of fluid (Keynes, 1923). This fluid does not escape because the nipple ducts are occluded by smooth muscle contraction, dried secretions, and keratinized epithelium. Breast fluid can be obtained by nipple aspiration in a significant proportion of women without spontanous nipple discharge with the use of a modified breast pump (Petrakis et al, 1975). This fluid contains several types of cells, including exfoliated breast epithelial cells (King et al, 1975). Because breast cancer develops from ductal and lobular epithelium, NAF is a potentially useful epidemiological and clinical research tool.

Wrensch et al (1992) evaluated NAF in a cohort of subjects with normal breast cancer risk. In this population, they demonstrated that subjects with NAF which contained normal cytology, hyperplasia without atypia, or atypical hyperplasia have a risk of breast cancer similar to subjects who have a biopsy with a similar diagnosis. We found that malignant (class IV) cells in NAF after excisional biopsy were 100% (7/7 specimens) and atypical (class III) cells 88% (7/8 specimens) predictive of the presence of residual breast cancer. NAF cytology, when comparing class III/IV cells with class I/II cells, was highly associated ($P$ = 0.001) with risk of residual cancer and with tumour size ($P$ = 0.004).

Computerized image analysis is able to quantitate the DNA index and cell cycle parameters on a cell by cell basis. The prognostic utility of this modality has been demonstrated for a variety of tumours, including breast cancer (Dressler et al, 1988). While flow cytometry is the standard method to determine cellular DNA, image analysis using Feulgen-stained cell preparations is gaining increased acceptance because of its ability to evaluate samples of relatively scant cellularity. DNA indices in epithelial cells from nipple aspirate specimens were determined and evaluated for their correlation with cytological class. In a prior study, we reported an association between both DNA index and % cells in G2/M and abnormal cytology. The association between these two markers and breast cancer risk was less clear, although the samples in the prior study were primarily from subjects with an intact breast. In NAF from mastectomy specimens, DNA index was significantly associated with residual cancer, large tumour size and atypical cytology. In addition, higher % cells in G2/M was significantly associated with residual cancer and large tumour size.

NAF cytology was highly specific (97% after excisional biopsy) in ruling out, but not highly sensitive (46% after needle biopsy, 36% after excisional biopsy) in detecting, residual cancer. There was one false positive, a specimen containing atypical cytology from a breast not found to contain residual cancer. Given that only approximately 1% of a mastectomy specimen is evaluated during routine histological review, it is unknown if this case is truly a false positive or rather the presence of residual cancer which was

© 1999 Cancer Research Campaign not identified on routine evaluation.

Although abnormal cytology was highly predictive of residual cancer, abnormal cytology was detected in only 36% of specimens with residual DCIS or invasive cancer. As such, while a finding of abnormal cytology is highly suggestive of the presence of residual cancer, normal cytology does not exclude the possibility that residual cancer exists. We therefore evaluated the two other markers (DNA index and % cells in G2/M) which were associated with residual cancer, in an attempt to increase our ability to predict if residual cancer exists. While neither marker increased the predictive ability of NAF cytology alone, we are currently evaluating other candidate markers, including insulin-like growth factor type 1 (IGF-1) and IGF binding protein type 3, to determine if these markers, when combined with cytology, will improve our ability to use NAF markers to predict residual cancer in the mastectomy specimen.

In summary, NAF can be obtained from mastectomy specimens and biological markers of breast cancer risk evaluated with a high degree of success. Both abnormal cytology and higher DNA index were associated with tumour progression. DNA index was associated with atypical cytology. Atypical cytology, DNA index and % cells in G2/M were each associated with residual cancer. Malignant cytology was 100% and atypical cytology 88% predictive of residual DCIS or invasive cancer after excisional biopsy. These findings provide insight into breast cancer tumour development and progression, and may prove useful in guiding therapy.

### REFERENCES

Agresti A (1990) *Categorical Data Analysis*, pp 59–64 and 321–324. Wiley & Sons: New York

Anscher MS, Jones P, Prosnitz LR, Blackstock W, Hebert M, Reddick R, Tucker A, Dodge R, Leight G Jr and Iglehart JD (1993) Local failure and margin status in early-stage breast carcinoma treated with conservation surgery and radiation therapy. *Ann Surg* **218**: 22–28

Dressler LG, Seamer LC, Owens MA, Clark GM and McGuire WL (1988) DNA flow cytometry and prognostic factors in 1331 frozen breast cancer specimens. *Cancer* **61**: 420–427

Earp HS, Dawson TL, Li X and Yu H (1995) Heterodimerization and functional interaction between EGF receptor family members: a new signaling paradigm with implications for breast cancer research. *Breast Cancer Res Treat* **35**: 115–132

Ellison DA, Maygarden SJ and Novotny DB (1995) Quantitative DNA analysis of fresh solid tumors by flow and image cytometric methods: a comparison using the Roche Pathology Workstation image analyzer. *Modern Pathol* **8**: 275–281

Falk RT, Dorgan JF, Kahle L, Potischman N and Longcope C (1996) Assay reproducibility of hormone measurements in postmenopausal women. *Cancer Epidemiol Biomarkers Prev* **6**: 429–432

Frazier TG, Wong RWY and Rose D (1989) Implications of accurate pathologic margins in the treatment of primary breast cancer. *Arch Surg* **124**: 37–38

Gwin JL, Eisenberg BL, Hoffman JP, Ottery FD, Boraas M and Solin LJ (1993) Incidence of gross and microscopic carcinoma in specimens from patients with breast cancer after re-excision lumpectomy. *Ann Surg* **218**: 729–734

Haagensen C, Lane N, Lattes R and Bodian C (1978) Lobular neoplasia of the breast. *Cancer* **421**: 737–769

Hallahan DE, Michel AG, Halpern HJ, Awan AM, Desser R, Bitran J, Recant W, Wyman B, Spelbring DR and Weichselbaum RR (1989) Breast conserving surgery and definitive irradiation for early stage breast cancer. *Int J Radiat Oncol Biol Phys* **17**: 1211–1216

Harris JR, Morrow M and Bonnadonna G (1993) Cancer of the breast. In: *Cancer: Principles and Practice of Oncology*, DeVita VT Jr, Hellman, S and Rosenberg SA (eds), 4th ed, pp. 1264–332. J.B. Lippincott: Philadelphia.

Keynes G (1923) Chronic mastitis. *Br J Surg* **11**: 89–121

King EB, Barrett D, King MC and Petrakis NL (1975) Cellular composition of the nipple aspirate specimen of breast fluid. I. The benign cells. *Am J Clin Pathol* **64**: 728–738

King EB, Chew KL, Petrakis NL and Ernster VL (1983) Nipple aspirate cytology for the study of breast cancer precursors. *J Natl Cancer Inst* **71**: 1115–1121

Lagios MD (1992) Pathologic features related to local recurrence following lumpectomy and irradiation. *Semin Surg Oncol* **8**: 122–128

Lai LC, Ghilchik MW, Shaikh NA, Reed MJ and James VHT (1989) Relationship of epidermal growth factor and dehydroepiandrosterone and its sulphate in breast cyst fluid. *Br J Cancer* **60**: 320–323

Leis HP (1991) Prognostic parameters for breast carcinoma. In: *The Breast*, Bland KI and Copeland EM (eds), pp. 331–351. WB Saunders: Philadelphia

Melegos DN, Diamandis EP, Oda H, Urade Y and Hayaishi O (1991) Immunofluorometric assay of prostaglandin D synthase in human tissue extracts and fluids. *Clin Chem* **42**: 1984–1991

McCormick B, Kinne D, Petrek J, Osborne M, Cox L, Shank B, Hellman S, Yahalom J and Rosen PP (1987) Limited resection for breast cancer: a study of inked specimen margins before radiotherapy. *Int J Radiat Oncol Biol Phys* **13**: 1667–1671

Olivotto IA, Rose MA, Osteen RT, Love S, Cady B, Silver B, Recht A and Harris JR (1989) Late cosmetic outcome after conservation surgery and radiotherapy: analysis of causes of cosmetic failure. *Int J Radiat Oncol Biol Phys* **17**: 747–753

Petrakis NL, Mason L, Lee R, Sugimoto B, Pawson S and Catchpool F (1975) Association of race, age, menopausal status, and cerumen type with brest fluid secretion in nonlactating women, as determined by nipple aspiration. *J Natl Cancer Inst* **54**: 829–834

Sauer R, Schauer A, Rauschecker HF, Schumacher M, Gatzemeier W, Schmoor C, Dunst J, Seegenschmiedt MH and Marx D (1992) Therapy of small breast cancer: a prospective study on 1036 patients with special emphasis on prognostic factors. *Int J Radiat Oncol Biol Phys* **23**: 907–914

Sauter ER, Daly M, Linahan K, Ehya H, Engstrom PF, Bonney G, Ross EA, Yu H and Dlamandis E (1996) Prostate-specific antigen levels in nipple aspirate fluid correlate with breast cancer risk. *Cancer Epidem Biomarkers Prev* **5**: 967–970

Sauter ER, Ross E, Daly M, Klein-Szanto A, Engstrom PF, Sorling A, Malick J and Ehya H (1997) Nipple aspirate fluid: a promising non-invasive method to identify cellular markers of breast cancer risk. *Br J Cancer* **76**: 494–501

Schnitt SJ, Connolly JL, Khettry U, Mazoujian G, Brenner M, Silver B, Recht A, Beadle G and Harris JR (1987) Pathologic findings on reexcision of the primary site in breast cancer patients considered for treatment by primary radiation therapy. *Cancer* **59**: 675–681

Schwartz GF, Rosenberg AL, Danoff BF, Mansfield CM and Feig SA (1984) Lumpectomy and level I axillary dissection prior to irradiation for “operable” breast cancer. *Ann Surg* **200**: 554–560

Stotter AT, McNeese MD, Ames FC, Oswald MJ and Ellerbroek NA (1989) Predicting the rate and extent of locoregional failure after breast conservation therapy for early breast cancer. *Cancer* **64**: 2217–2225

Thompson DMP, Krupey J, Freeman SO and Gold P (1969) The radioimmunoassay of circulating carcinoembryonic antigen of the human digestive system. *Proc Natl Acad Sci USA* **64**: 161–167

Veronesi U, Volterrani F, Luini A, Saccozzi R, DelVecchio M, Zucali R, Galimberti V, Rasponi A, Di Re E and Squicciarini P (1990) Quadrantectomy versus lumpectomy for small size breast cancer. *Eur J Cancer* **26**: 671–673

Wrensch MR, Petrakis NL, King EB, Miike R, Mason L, Chew KL, Lee MM, Ernster VL, Hilton JF and Schweitzer R (1992) Breast cancer incidence in women with abnormal cytology in nipple aspirates of breast fluid. *Am J Epidem* **135**: 130–141

Yeatman TJ and Bland Kl. Staging of breast cancer. In: *The Breast*, Bland KI and Copeland EM (eds), pp. 313–330. WB Saunders: Philadelphia

*© 1999 Cancer Research Campaign*
